# Mammillary body atrophy and other MRI correlates of school-age outcome following neonatal hypoxic-ischemic encephalopathy

**DOI:** 10.1038/s41598-021-83982-8

**Published:** 2021-03-03

**Authors:** Kim V. Annink, Linda S. de Vries, Floris Groenendaal, Rian M. J. C. Eijsermans, Manouk Mocking, Monique M. J. van Schooneveld, Jeroen Dudink, Henrica L. M. van Straaten, Manon J. N. L. Benders, Maarten Lequin, Niek E. van der Aa

**Affiliations:** 1 Department of Neonatology, UMC Utrecht Brain Centre, Wilhelmina Children’s Hospital, University Utrecht, Internal Room Number KE04.123.1, Lundlaan 6, 3508AB Utrecht, The Netherlands; 2 Child Development and Exercise Centre, UMC Utrecht, Utrecht, The Netherlands; 3 Department of Paediatric Psychology and Social Work, UMC Utrecht, Utrecht, The Netherlands; 4 Department of Neonatology, Isala Clinics, Zwolle, The Netherlands; 5 Department of Radiology, UMC Utrecht, Utrecht, The Netherlands

**Keywords:** Hypoxic-ischaemic encephalopathy, Outcomes research, Predictive markers, Paediatric research, Hypoxia, Neonatal brain damage, Imaging techniques, Learning and memory, Intelligence, Neuroscience, Cognitive neuroscience

## Abstract

The mammillary bodies (MB) and hippocampi are important for memory function and are often affected following neonatal hypoxic ischemic encephalopathy (HIE). The aim of this study was to assess neurodevelopmental outcome in 10-year-old children with HIE with and without therapeutic hypothermia. Additional aims were to assess the associations between MB atrophy, brain volumes (including the hippocampi), white matter microstructure and neurodevelopmental outcome at school-age. Ten-year-old children with HIE were included, who were treated with therapeutic hypothermia (n = 22) or would have qualified but were born before this became standard of care (n = 28). Children completed a neuropsychological and motor assessment and MRI. Mammillary bodies were scored as normal or atrophic at 10 years. Brain volumes were segmented on childhood MRI and DTI scans were analysed using tract-based spatial statistics. Children with HIE suffered from neurocognitive and memory problems at school-age, irrespective of hypothermia. Hippocampal volumes and MB atrophy were associated with total and performance IQ, processing speed and episodic memory in both groups. Normal MB and larger hippocampi were positively associated with global fractional anisotropy. In conclusion, injury to the MB and hippocampi was associated with neurocognition and memory at school-age in HIE and might be an early biomarker for neurocognitive and memory problems.

## Introduction

Infants with hypoxic-ischemic encephalopathy (HIE) due to presumed perinatal asphyxia are at risk for death, motor problems, cognitive and memory deficits, behavioural problems and epilepsy^1,2^. Infants with moderate to severe HIE are treated with whole body hypothermia. This has shown to improve 18–24 month survival without neurological disabilities and to reduce cerebral palsy (CP) and epilepsy at school-age^1,3–6^. However, a considerable number of children treated with hypothermia still has neurocognitive problems at school-age^5,7^.

To understand and adequately predict neurodevelopmental problems at school-age in children with HIE treated with hypothermia, it is important to elucidate which brain areas are injured and contribute to long-term problems. Brain injury can be diagnosed with the use of different MRI techniques, e.g. by differences in signal intensity on conventional imaging, measuring volumes of brain structures/regions and determining the microstructure of white matter tracts using diffusion tensor imaging (DTI). Previous studies in children with HIE not treated with hypothermia, showed that smaller hippocampal volumes were associated with a lower IQ and memory problems^8,9^. Furthermore, it was recently hypothesized that atrophy of the mammillary bodies (MB) at three months of age might be predictive of childhood outcome^10^. Similar to the hippocampus, MB are known to be important for memory function^11^.

In this study we examined the association between MB atrophy, brain volumes and white matter microstructure at 10 years of age and neurodevelopmental outcome in children with a history of HIE with and without therapeutic hypothermia. The first aim was to assess motor and neurocognitive outcome at 10 years of age in children treated with and without hypothermia. Secondly, the association between MB atrophy and neurodevelopmental outcome was assessed. Thirdly, the association between childhood subcortical, cortical and white matter brain volumes and neurodevelopmental outcome were investigated, also in combination with MB atrophy. The final aim was to determine the association between white matter microstructure and neurodevelopmental outcome at school-age.

## Materials and methods

### Study population

For this observational study, children were eligible if they: (1) were born at a gestational age ≥ 36 weeks, (2) had been admitted to the neonatal intensive care unit of the UMC Utrecht or Isala Clinics between 2006 and 2009 because of acute perinatal asphyxia, (3) (would have) qualified for therapeutic hypothermia and (4) had reached the age of ten years. Exclusion criteria were congenital brain abnormalities and/or other (chromosomal/metabolic) anomalies, acquired brain injury due to trauma or infection, severe cerebral palsy making MRI and neurodevelopmental testing impossible and contraindications for MRI, such as braces, a pacemaker or claustrophobia.

Children born in the year before therapeutic hypothermia became standard of care in 2008, who met the therapeutic hypothermia criteria, were included in the non-hypothermia group (non-HT group). Children that were treated with hypothermia in the year after therapeutic hypothermia became standard of care were included in the hypothermia group (HT group). Besides hypothermia there were no (major) changes in clinical care for infants with HIE. Outcome was assessed in both groups, but assessing efficacy of HT was not the aim of this study.

Written parental informed consent and child assent was obtained for all study participants. The study was conducted according to the declaration of Helsinki and the national regulations. The medical ethical committee of the UMC Utrecht approved the study (NL44807.041.14).

### Neonatal clinical and MRI data

Baseline characteristics and standard follow-up data were retrospectively collected from the electronic patient files, as well as the neonatal MRI scans if these were available. Neonatal MRI scans were conducted on a 1.5 T in all infants in the non-HT group and partly in the HT group or 3.0 T MR scan from 2009 onwards in the HT group (Philips Healthcare, Best, the Netherlands). The scan protocols included diffusion Weighted Imaging (DWI), Inversion recovery or T1-weighted imaging and T2-weighted imaging, as was reported previously^12^.

When available, the neonatal MRI scans were retrospectively scored according to Weeke et al*.* by a neonatologist and neuroradiologist who were blinded to childhood outcome and reached consensus for each scan^12^. This MRI score includes 17 brain structures that are scored 0–2 based on diffusion and signal intensity, the higher the score the more injury. Injury to the MB on the neonatal MRI was assessed separately. MB were classified as normal, equivocal or abnormal based on neonatal T1- and T2-weighted sequences and DWI. The definition of normal was a similar signal intensity of the MB as the surrounding tissue and no swelling. The MB were scored “equivocal” if there appeared to be a higher signal intensity of the mammillary bodies on the T2-weighted image or a low signal on T1-weighted imaging but without swelling, so it was uncertain if this could be a partial volume effect. Abnormal was defined as an increased signal intensity of the mammillary bodies on the T2-weighted image, a low signal on T1-weighted imaging or diffusion restriction on DWI, in combination with swelling of the MB^10^.

### Neurocognitive tests at 10 years of age

All children completed a full neuropsychological assessment performed by a paediatric neuropsychologist. Intelligence was tested with the Wechsler Intelligence Scale for Children, Third edition (WISC-III-NL)^13^. Total IQ (TIQ), Verbal IQ (VIQ) and Performance IQ (PIQ) were derived from the WISC-III-NL. The Processing Speed Index of the WISC-III-NL was used to evaluate the speed of information processing^13^. The verbal long-term memory was tested with the 15-words test^14^, which consists of five learning trials with immediate recall of words (resulting in a total score) and a delayed recall after 25 min (resulting in a delayed score). Verbal Working Memory was measured with the Digit Span task of the WISC-III-NL^13^. Visual-spatial Working Memory was tested with the spatial span task of the Wechsler nonverbal scale of ability^15^. Visual-spatial long-term memory was assessed with the Rey Complex Figure Test^16^. To test attention, the subtests Sky Search, Score!, Creature Counting and Sky Search DT of the Test of Everyday Attention of Children were administered^17^.

Neurocognitive test results are presented in norm, decile or t-scores depending on the test and specified in the tables.

### Motor assessment at 10 years of age

Motor performance was assessed with the Bruininks-Oseretsky Test of Motor Proficiency second edition (BOT-2). The BOT-2 scores four different domains: fine motor manual control, manual coordination, body coordination and strength and agility^21^. Both a total composite score as composite scores of the four domains were used. Based on standard composite scores children are scored as well above average (above + 2 SD), above average (+ 1 SD until + 2 SD, average (− 1 SD until + 1 SD, below average (− 1 SD until − 2SD) and well below average (below − 2 SD).

### MRI at 10 years of age

MRI scans were conducted at a 3.0 T MRI scanner without sedation (Philips Healthcare, Best, the Netherlands). The scan protocol contained among others 3D-T1-weighted imaging (TE = 4.6 ms; TR = 10 ms; 180 0.8-mm slices, field of view 240 × 240 mm, 304 × 304 matrix), fluid attenuated inversion recovery (FLAIR) (TE = 120 ms; TR = 10000 ms; 26 4-mm slices, field of view 230 × 182 mm, 352 × 272 matrix ) and DWI (TE = 96; TR = 3317, 66 2-mm slices, field of view 224 × 224 mm, 112 × 112 matrix; using 13 non-diffusion weighted images and 111 diffusion weighted images with b-values of 500 s/mm^2^ (15), 1000 s/mm^2^ (32) and 2000s/mm^2^ (64)). In addition, a single non-diffusion weighted image was acquired in the opposite phase-encoding direction.

### MRI-scoring at 10 years of age

The 3D-T1-weighted and FLAIR images at 10 years of age were scored according to the scoring method used by van Kooij et al*.:* (1) no injury, (2) solitary white matter lesions and/or (focal) thinning of the corpus callosum, (3) watershed injury, (4) deep grey matter injury or (5) focal infarction^22^. The childhood scans were scored by two readers that reached consensus for all patients.

**Figure 1:**
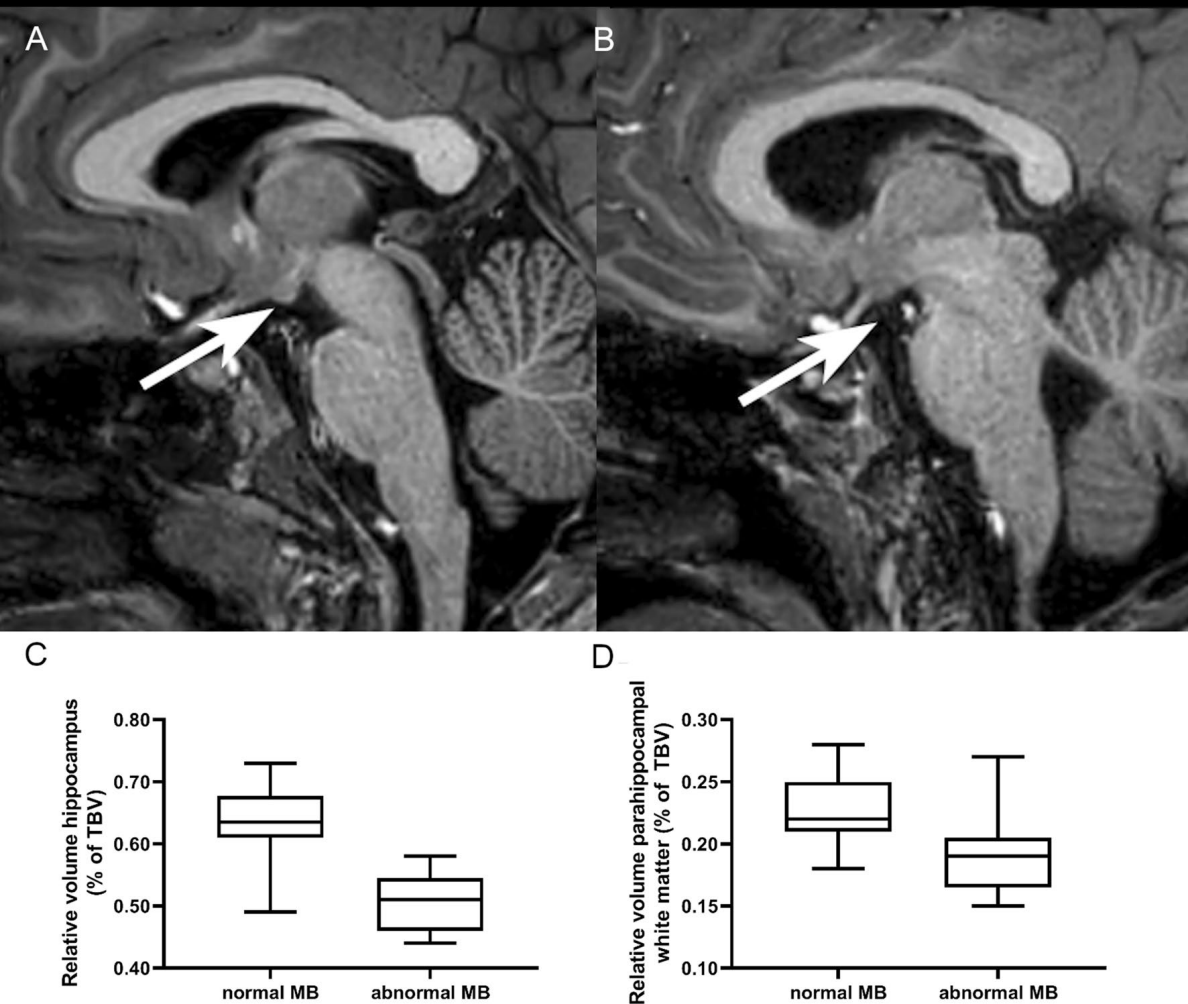
Example of normal MB (**A**) and atrophy of the MB (**B**) at 10 years of age. Differences in relative hippocampal volume (**C**) and parahippocampal volume (**D**) between children with normal MB and atrophy of the MB at 10 years of age.

Mammillary bodies at 10 years of age were scored based on the sagittal plane of the 3D-T1-weighted MRI. The mammillary bodies were categorized as normal if there was no atrophy or if they appeared smaller but were still detectable. Mammillary bodies were scored as atrophic if they were not visible (Fig. 1). Two authors scored the MRI scans together, both were blinded for outcome. A third blinded reader scored 18 random patients to determine the interrater agreement.

### MRI-volumetric measurements at 10 years of age

Volumetric segmentation of 3D-T1-weighted MR scans was performed using Freesurfer version 6.0.0^23^. Segmentation of the subcortical deep grey matter structures and parcellation of the white matter and cortex was based on signal intensity differences. Images with large movement artefacts were excluded. This resulted in segmentation of 20 subcortical volumes and parcellation of 40 white matter and 40 cortical volumes. MRI scans with topological defects, skull strip errors or white matter errors were manually corrected prior to segmentation. For more elaborative details of this method, we refer to earlier publications^23^. Total volumes refer to the uncorrected volume in ml and relative volumes to the volume of the structure as a percentage of total brain volume (TBV) excluding cerebral spinal fluid.

### MRI-TBSS at 10 years of age

Image processing and analysis of the DWI were performed using tools from the FMRIB's Software Library (FSL v6.0.3)^24^, DTI-ToolKit (DTI-TK) (v.2.3.1) and tract-based spatial statistics (TBSS v1.2)^25^. FSL was used to correct for susceptibility induced distortions, using the non-diffusion weighted images with opposite-phase directions. Next, the data were corrected for eddy-current induced distortions and head movements followed by a brain extraction to remove non-brain tissue. Finally, the tensor was fitted, after which all data were normalized to a template. DTI-TK, which uses a tensor based registration, was used to create a population specific template and to normalize all data. A mean Fractional Anisotropy (FA) map was derived from this template to create a mean FA skeleton. Using TBSS, a FA threshold of ≥ 0.15 was used to limit the inclusion of non-white matter voxels and voxels with high-inter subject variability. The FA values of each subject were projected on this skeleton for further analysis. A generalized linear model was used to assess the relationship between FA and all motor and neuropsychological variables, hippocampal volume and mammillary body atrophy. Analyses were performed using Randomise and were subject to family-wise-error correction for multiple comparisons following threshold-free cluster enhancement and *p*-values < 0.05 were considered significant.

### Statistical analysis

Statistical analysis was performed using SPSS version 25 (IBM corp., Armonk NY, United States).

First the baseline characteristics were compared between the non-HT versus HT group to assess whether these groups were comparable. Afterwards neurocognitive and motor outcome was determined in the total group and compared between the non-HT and HT group. For both analyses the independent t-test or Mann–Whitney-U-test for continuous variables. For categorical variables the Chi^2^-test was used.

Secondly, the association between MB atrophy and neurodevelopmental outcome was assessed. Neurodevelopmental outcome measures and baseline characteristics were compared between infants with and without MB atrophy on their MRI at 10 year using the independent t-test or Mann–Whitney-U-test depending on the Gaussian distribution. Univariate linear regression analysis was performed with the outcome measure as dependent variable and MB atrophy score as independent variable. The agreement between neonatal MB body abnormalities and childhood MB atrophy was also assessed.

Thirdly, the association between childhood subcortical, cortical and white matter brain volumes and neurodevelopmental outcome were investigated. Univariate linear regression analysis was performed with the outcome measure as dependent variable and the brain volume as independent variable. Non-significant associations were excluded for the following analysis.

Afterwards, to determine whether there was multicollinearity, brain volumes were compared between infants with and without MB atrophy using the independent t-test or Mann–Whitney-U-test depending on the Gaussian distribution. Also, correlations between the different brain volumes were calculated with Pearson’s correlation or Spearman’s correlation test, depending on the normality.

Due to the multicollinearity, multivariable logistic regression was performed with a binary outcome measure (neurocognitive/motor test result <− 1 SD) as dependent variable and total hippocampal volume or MB atrophy, total white matter, total grey matter, total lateral ventricle and total cerebellar volume as independent variables regarding multicollinearity. This analysis was performed to take other brain structures into account when assessing the effect of either hippocampal volume or MB atrophy on outcome. Variables with a *p*-value < 0.05 were retained in the model and those with a *p*-value ≥ 0.1 deleted.

Finally TBSS analysis was performed using a generalized linear model to determine the association between global FA values and neurodevelopmental at school-age, and between hippocampal volumes or MB atrophy and global FA values. All analysis were corrected for sex and age at scan.

In general, *p*-values < 0.05 were considered statistically significant. However, *p*-values were corrected for multiple comparison by dividing 0.05 by the number of brain volumes or tests. The significant *p*-values, corrected for multiple comparisons, are reported in the footnotes of the tables.

## Results

### Neonatal and childhood characteristics

**Figure 2:**
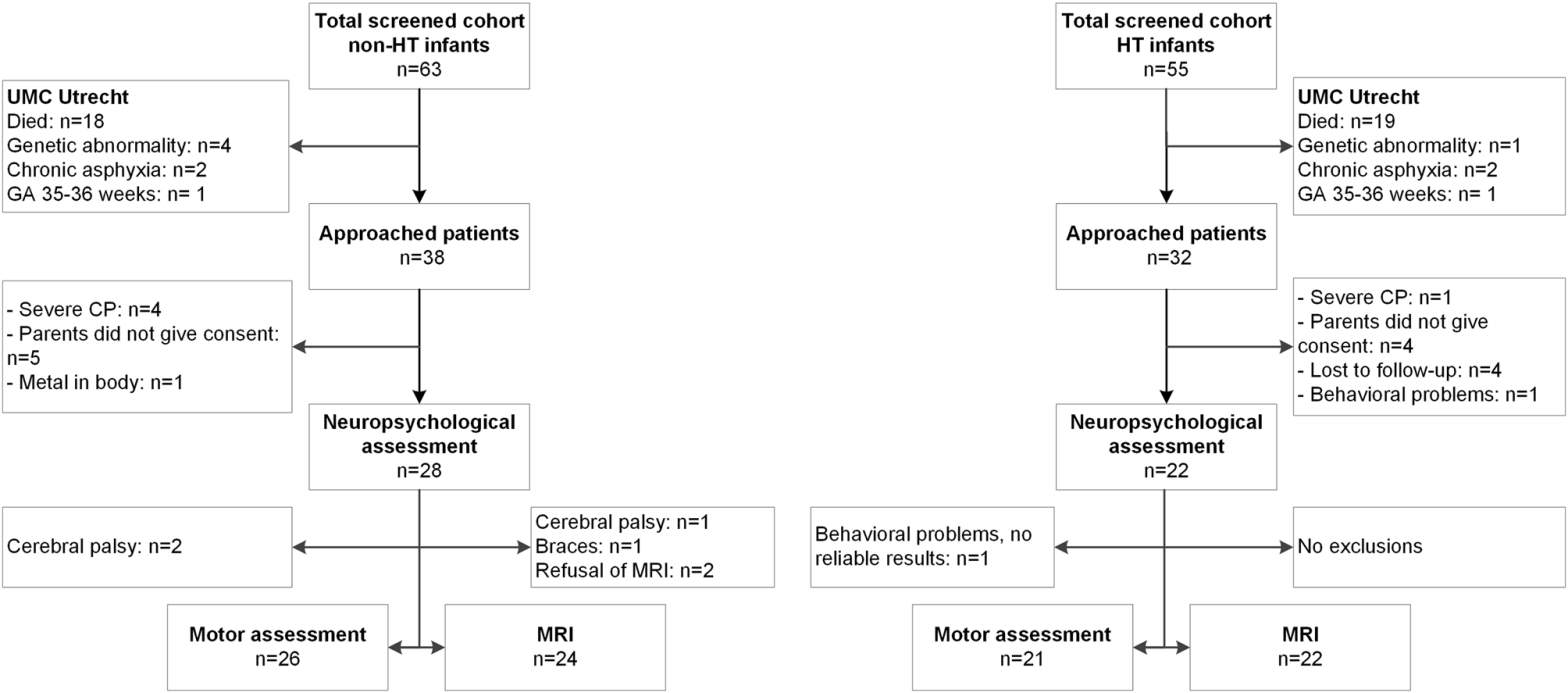
Flow chart of inclusion and exclusion for the children in the non-HT group (left) and for the children in the HT group (right). For Isala Clinics only data were available from the approached children.

Fifty patients were included in the study. For the non-HT group 38 patients were approached and 28 were included (72%). For the HT group 32 patients were approached and 22 were included (69%). See Fig. 2 for the flowchart. In the non-HT group 6 of 38 approached patients (16%) had CP and in the HT group 1 of the 32 approached patients (3%), most of them were too severely affected to take part in the study.

Table 1 shows the neonatal and childhood characteristics.

**Table 1:** Neonatal and childhood characteristics.

Characteristic	HIE without hypothermia (n = 28)	HIE with hypothermia (n = 22)	*p*-value
Male, n (%)	17 (60.7)	15 (68.2)	0.59
Gestational age in weeks, median (IQR)	40.86 (39.50–41.61)	40.21 (39.68–41.50)	0.53
Birth weight in gram, median (IQR)	3485 (3103—3700)	3710 (3280–4410)	0.009 *
Apgar 1 min, median (IQR)	2 (2–4)	1 (1–2)	0.006*
Apgar 5 min, median (IQR)	4 (3–6)	3.5 (3–4.75)	0.18
Apgar 10 min, median (IQR)	6 (4.5–7.5)	5 (4–6)	0.18
Highest lactate within first 12 h after birth, median (IQR)	7.7 (3.65–11.13)	12.4 (5.73–19.13)	< 0.05*
Lowest pH within first 12 h after birth, median (IQR)	7.14 (6.99–7.34)	6.99 (6.78–7.23)	0.06
**Neonatal seizures, n (%)**			0.78
No	5 (17.9)	5 (22.7)	
Yes, subclinical	5 (17.9)	5 (22.7)	
Yes, clinical	18 (64.3)	12 (54.5)	
**Neonatal worst aEEG background pattern, n (%)**			0.05
Normal (continuous normal voltage (+ sleep wake cycling), discontinuous low voltage)	9 (75)	6 (38)	
Abnormal (Burst suppression, continuous low voltage, flat trace)	3 (25)	10 (63)	
**Neonatal MRI, n (%)**			0.01*
No	7 (25)	0 (0)	
Yes	21 (75)	22 (100)	
Postnatal age at MRI in days, median (IQR)	4 (3–6)	5 (4–8)	0.004*
Total neonatal MRI score (total cohort), median (IQR)	8 (4.5–17.5)	4 (0–11)	0.03*
Total neonatal MRI score (Utrecht), median (IQR)	8.5 (6.5–19.0)	2.0 (0.0–12.5)	0.01*
Education mother**, median (IQR)	6 (5–6)	6 (5–6)	0.82
Education father**, median (IQR)	5 (5–6)	5 (5–6)	0.69
Age at follow-up study in months, median (IQR)	126 (125–127)	125 (123.8–127)	0.12
Weight in kg at 10 years of age, median (IQR)	34.8 (31.0–40.8)	38.4 (34.3–44.9)	0.14
Length in cm at 10 years of age, median (IQR)	145.0 (141.3–148.0)	146.0 (142.1–149.5)	0.16
Head circumference in cm at 10 years of age, median (IQR)	53.5 (51.5–54.2)	54.1 (53.0–54.5)	0.07
**Grade repetition or skipping, n (%)**			0.42
No	21 (75)	18 (81.8)	
Grade repetition	7 (25)	3 (13.6)	
Grade skipping	0 (0)	1 (4.5)	
**Epilepsy at 10 years of age, n (%)**			0.41
Never	23 (82.1)	19 (86.4)	
In the past	3 (10.7)	2 (9.1)	
At present, without medication	0 (0)	1 (4.5)	
At present, with medication	2 (7.1)	0 (0)	
**Audiovisual problems at 10 years of age, n (%)**			0.46
No	17 (60.7)	17 (77.3)	
Visual problems	9 (32.1)	4 (18.2)	
Hearing problems	2 (7.1)	1 (4.5)	
**MRI at 10 years of age, n (%)**			0.20
No injury	8 (33)	8 (36)	
Solitary white matter lesions and/or (focal) thinning of the corpus callosum	13 (54)	9 (41)	
Watershed injury	1 (4)	2 (9)	
Deep grey matter injury	8 (8)	0 (0)	
Focal infarction	0 (0)	3 (14)	

*p-value < 0.05.

**SES according to Verhage classification.

The median birthweight was significantly lower in the non-HT group compared to the HT group. The median Apgar at 1 min after birth was significantly lower in the HT group and the lactate significantly higher, which suggests that the HT group had more severe perinatal asphyxia.

At school-age, though not significantly different, in the non-HT group 7.1% of the children had epilepsy with anticonvulsive medication and none in the HT group. In the non-HT group 60.7% had no hearing or visual problems compared to 77.3% in the HT group.

The total neonatal MRI score of Weeke et al*.* was significantly higher in the non-HT group compared to the HT group, but significantly fewer neonatal MR scans were performed in this group as an MRI was only performed in Isala Clinics in the pre-hypothermia era if brain injury was suspected. In the UMC Utrecht all patients underwent an MRI, analysis of this subgroup showed also higher neonatal MRI scores in the non-HT group.

The MRI score used by van Kooij et al*.* at 10 years of age was comparable between the HT (median 2 (range 1–5) and non-HT group (median 2, (range 1–4) *p* = 0.20). At 10 years of age, atrophy of the MB was present in 17% in the non-HT group and 50% in the HT group**.**

### Neurodevelopmental outcome

Table 2 shows the motor and neuropsychological outcomes of the children in the non-HT and HT group. There were no differences in behavioural problems between the non-HT and HT group reported in CBCL and BRIEF questionnaires by parents or teachers^18–20^.

**Table 2:** Neurodevelopmental outcome.

Neurodevelopmental outcome	HIE without hypothermia (n = 28)	HIE with hypothermia (n = 21)	*p*-value*
**Intelligence**			
Total intelligence quotient, mean (SD)	94.3 (15.4)	94.1 (17.1)	0.96
Verbal intelligence quotient, mean (SD)	97.7 (12.8)	98.8 (15.5)	0.78
Performance intelligence quotient, mean (SD)	94.0 (15.7)	90.3 (18.4)	0.46
Processing Speed, mean (SD)	102.3 (15.9)	91.1 (14.2)	0.02
**Memory**			
Verbal long-term memory-immediate recall, mean decile score (SD)	4.7 (3.0)	2.2 (2.3)	0.001**
Verbal long-term memory-delayed recall, mean decile score corrected for age (SD)	3.5 (2.7)	2.2 (2.2)	0.03
Verbal Working Memory, mean norm score (SD)	9.3 (4.1)	9.3 (2.8)	0.97
Visual-spatial Working Memory, mean t-score (SD)	47.7 (9.5)	48.5 (14.1)	0.84
Visual-spatial long-term memory-direct recall, mean t-score (SD)	39.2 (13.8)	32.7 (15.2)	0.12
Visual-spatial long-term memory-delayed recall, mean t-score (SD)	37.0 (11.9)	31.1 (13.9)	0.11
**Attention**			
Sustained attention, mean (SD)	8.2 (3.1)	8.7 (3.4)	0.55
Selective attention, mean (SD)	9.8 (2.4)	9.4 (2.6)	0.59
**Motor outcome**			
Total composite score, mean (SD)	48.6 (11.1)	43.0 (9.0)	0.06
Fine Manual Control standard score, mean (SD)	45.5 (9.9)	41.2 (9.3)	0.09
Manual Coordination standard score, mean (SD)	48.9 (8.5)	45.0 (11.1)	0.19
Body coordination standard score, mean (SD)	43.0 (8.5)	40.3 (9.2)	0.32
Strength and agility standard score, mean (SD)	57.6 (9.9)	52.6 (8.2)	0.07

*For the outcomes of the neuropsychological assessments, after correction for multiple comparison, *p*-values < 0.004 were statistically significant. For the motor outcome, *p*-values < 0.01 were statistically significant.

**Statistically significant.

In the total group, 36.7% of the children scored below − 1SD on the BOT-2. In the non-HT group, two infants had CP and could not be tested on the BOT-2; they were considered to have an abnormal score. Two of the children in the HT group, had impaired function of one arm because of obstetric brachial plexus injury.

In the total cohort, TIQ was below − 2SD in 6.3% of the children and below − 1SD in 29.2% of the children. VIQ was below − 2SD in 2.1% and below − 1SD in 21.3% of the children. PIQ was below − 2SD in 8.7% and below -1SD in 37.0% of the children. The processing speed was below − 2SD in 2.1% and below − 1SD in 22.9% of the children.

### MRI-MB atrophy and neurodevelopmental outcome

In the whole study group, 44 patients had a scan at 10 years of age that was considered to be of sufficient quality to score the MB, showing atrophy in 38%. Birth weight, Apgar scores and pH did not differ between children with normal MB and atrophy of the MB at 10 years of age. Children with abnormal MB did have higher lactate levels compared to children with normal MB (13.6 vs 8.9 mmol/L, *p* = 0.04). A trend towards increased neonatal MRI scores according to Weeke et al*.* was seen in the children with MB atrophy, but this did not reach significance.

Table 3 shows the differences in outcome measures between infants with normal and abnormal MB. MB atrophy was significantly associated with lower TIQ, VIQ, PIQ, verbal long-term memory and visual spatial long-term memory scores, see also Supplementary Table 2. In total, 39 patients had a neonatal MRI scan as well as an MRI scan at 10 years of age.

**Table 3:** Neurodevelopmental outcome and MB at follow-up.

Test	Normal MB at 10y (n = 30)	Atrophy MB at 10y (n = 14)	*p*-value	Mean difference (95%CI)
**Intelligence**				
Total intelligence quotient, mean (SD)	99.9 (13.5)	80.6 (11.3)	< 0.001*	19.3 (10.8—27.7)
Verbal intelligence quotient, mean (SD)	101.8 (13.3)	87.6 (8.9)	0.002*	14.2 (5.4—23.0)
Performance intelligence quotient, mean (SD)	98.0 (14.2)	76.6 (10.5)	< 0.001*	21.4 (12.2—30.6)
Processing Speed, mean (SD)	100.9 (16.7)	86.7 (10.5)	0.007	14.2 (4.0—24.3)
**Memory**				
Verbal long-term memory-immediate recall, mean decile score (SD)	4.4 (2.8)	1.1 (0.3)	< 0.001*	3.3 (2.3—4.4)
Verbal long-term memory-delayed recall, mean decile score corrected for age (SD)	3.6 (2.7)	1.0 (0.0)	< 0.001*	2.6 (1.6–3.6)
Verbal Working Memory, mean norm score (SD)	9.3 (3.3)	9.1 (4.1)	0.90	0.1 (− 2.2–2.5)
Visual-spatial Working Memory, mean t-score (SD)	48.2 (12.0)	47.0 (12.2)	0.76	1.2 (− 6.8–9.3)
Visual-spatial long-term memory direct recall, mean t-score (SD)	40.8 (14.3)	22.7 (4.9)	< 0.001*	18.2 (12.2–24.1)
Visual-spatial long-term memory delayed recall, mean t-score (SD)	39.9 (11.5)	20.5. (2.4)	< 0.001*	19.4 (14.8–24.0)
**Attention**				
Sustained attention, mean (SD)	8.3 (3.1)	8.2 (3.7)	0.95	0.9 (− 2.1–2.3)
Selective attention, mean (SD)	10.0 (2.1)	8.5 (3.0)	0.11	1.5 (− 0.1–3.1)
**Motor outcome**				
Total composite score, mean (SD)	47.9 (10.0)	38.9 (7.1)	0.004*	9.0 (3.0–14.0)
Fine Manual Control standard score, mean (SD)	46.5 (9.1)	35.6 (5.9)	< 0.001*	10.9 (5.5–16.3)
Manual Coordination standard score, mean (SD)	49.2 (9.0)	41.2 (10.0)	0.01	8.0 (1.9–14.0)
Body coordination standard score, mean (SD)	42.6 (8.1)	36.9 (7.1)	0.03	5.7 (0.7–10.8)
Strength and agility standard score, mean (SD)	56.6 (9.5)	51.3 (8.7)	0.08	5.3 (− 0.7–11.4)

*For the outcomes of the neuropsychological assessments, after correction for multiple comparison, *p*-values < 0.004 were statistically significant. For the motor outcome, *p*-values < 0.01 were statistically significant.

The majority (91%) of the infants with normal neonatal MB also had normal MB at 10 years, while 76% of the infants with abnormal neonatal MB had atrophy at 10 years of age. The majority (90%) of the infants with equivocal MB on their neonatal scan, had normal MB at 10 years (Supplementary Table 1). The postnatal age at the moment of the neonatal MRI did not differ between infants with normal, equivocal or abnormal MB (*p* = 0.33).

The third reader on MB scored the same as the others in 83% of the childhood cases. In the three cases where the score was different, the MB was still present but smaller than usual.

### Univariate logistic regression: brain volumes versus neurodevelopmental outcome

Forty-one children (82%) had a 3D-T1-weighted sequence at 10 years of age of sufficient quality for segmentation. In the univariate linear regressions with the brain structure as independent variable and the neuropsychological test score as dependent variable, MB and relative hippocampal volume were significantly associated with multiple neuropsychological tests after correcting for multiple comparisons. The parahippocampal white matter and caudal anterior cingulate cortex were, after correcting for multiple comparisons, significantly associated with processing speed. All structures that showed an association (uncorrected *p*-value < 0.05) with one of the outcome measures are reported in supplementary Tables 2 and 3.

### Multivariable logistic regression: brain volumes versus neurodevelopmental outcome

Based on the results above, the association between atrophy of the MB and hippocampi and intelligence and memory appeared to be most relevant. These structures are both part of the Papez circuit, which is known to have a significant role in both memory and emotional expressions. Besides the MB and hippocampi, the anterior thalamus, parahippocampal and cingulate white matter are also part of this circuit and therefore multivariable logistic regression analyses were performed including all these structures.

All volumes assessed by Freesurfer were compared between infants with and without MB atrophy. After correction for multiple comparisons, children with MB atrophy had smaller relative hippocampal volumes (*p* < 0.001) and smaller relative parahippocampal white matter (*p* = 0.001) (Fig. 1). The other relative subcortical, white matter and cortical relative volumes did not differ between the groups.

Brain volumes of the Papez circuit were correlated with each other (data not shown), therefore it was impossible to include the different brain volumes in one model because of multicollinearity.

To correct for other brain volumes, we therefore made two different models. First, multivariable logistic regression was performed with the neuropsychological test results as dependent variable and MB atrophy, total white matter, total grey matter, total volume lateral ventricles and total cerebellar volume as independent variables. In this model MB atrophy was significantly associated with impaired TIQ, PIQ, VIQ, processing speed and visual-spatial long-term memory and verbal long-term memory. Secondly, we performed the same analyses but with total hippocampal volume instead of MB atrophy (Table 4). This showed that smaller hippocampal volumes were significantly associated with impaired TIQ, PIQ, processing speed and visual-spatial long-term memory and verbal long-term memory.

**Table 4:** Multivariable logistic regression models for different neuropsychological tests with MB and with total hippocampal volume.

Outcome measure	Model with MB	Odds ratio (95%CI)	Model with hippocampus	Odds ratio (95%CI)
**Cognition**				
Total intelligence quotient	MB atrophy	35.00 (5.53–221.39)	Hippocampus	0.19 (0.07–0.55)
Verbal intelligence quotient	Grey matter	0.98 (0.97–1.00)	White matter	0.97 (0.95–1.00)
	MB atrophy	7.26 (1.1–48.33)		
Performance intelligence quotient	MB atrophy	32.50 (5.12–206.16)	Hippocampus	0.19 (0.06–0.53)
Processing Speed	MB atrophy	3.75 (0.90–15.66)	Hippocampus	0.48 (0.23–0.99)
**Memory**				
Verbal long-term memory-immediate recall	MB atrophy	50.00 (5.40–463.20)	Hippocampus	0.24 (0.09–0.65)
			Lateral ventricles	1.2 (0.99–1.40)
Verbal long-term memory-delayed recall	MB atrophy	n/a^a^	Hippocampus	0.16 (0.05–0.47)
			Grey matter	1.02 (1.00–1.03)
Verbal Working Memory	Grey matter	0.99 (0.98–1.00)	Grey matter	0.99 (0.98–1.00)
	Lateral ventricles	1.11 (0.99–1.26)	Lateral ventricles	1.11 (0.99–1.26)
Visual-spatial Working Memory	n.s	n.s	n.s	n.s
Visual-spatial long-term memory direct recall	MB atrophy	n/a^a^	Hippocampus	0.33 (0.14–0.77)
Visual-spatial long-term memory delayed recall	MB atrophy	n/a^a^	Hippocampus	0.21 (0.07–0.63)

^a^No Odds Ratio’s could be calculated, because none of the infants with atrophy of the MB had a normal outcome.

### MRI-TBSS

**Figure 3:**
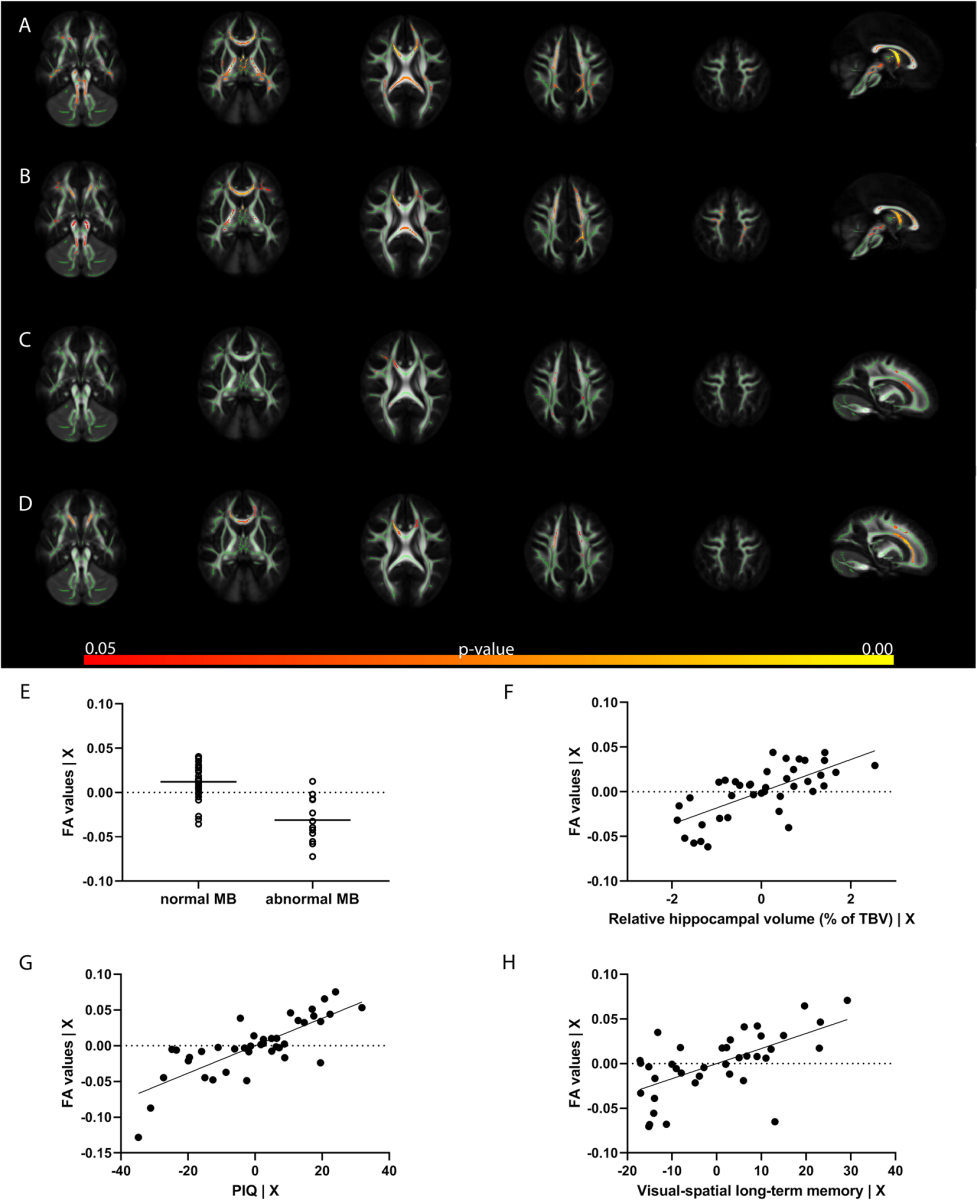
TBSS results. Results of TBSS analyses with five axial views and one midsagittal view for the MB (**A**) and hippocampus (**B**) and a parasagittal view for the neurocognitive and memory measures (**C**, **D**). All analyses were corrected for age and sex. This figure shows the associations between FA values and MB (**A**, **E**), relative hippocampal volume (**B**, **F**), PIQ (**C**, **G**) and visual-spatial long-term memory (delayed recall) (**D**, **H**). In the plots the unstandardized residuals for the FA values corrected for age and sex are visualized on the y axis, and the MB (**E**) or the unstandardized residuals for relative hippocampal volume (**F**), PIQ (**G**) or visual-spatial long-term memory (**H**) corrected for age and sex on the x-axis.

Given the observed association between the hippocampal volume and MB atrophy and neuropsychological outcome, these parameters were used for TBSS. TBSS analyses showed that larger hippocampal volumes, corrected for age and sex, had a wide spread positive association with FA-values throughout the brain. Children with normal MB had higher white matter FA values compared to infants with MB atrophy, after correction for age and sex. For the neurodevelopmental measures, only PIQ and visual-spatial long-term memory delayed recall had a significant positive association with FA values in the TBSS analyses. Age and sex were not associated with FA in any of the analyses. See Fig. 3.

## Discussion

This study shows that children at 10 years of age with a history of HIE due to presumed perinatal asphyxia have long-term neurodevelopmental problems. This also applied to children who were treated with therapeutic hypothermia. Parts of the Papez circuit, i.e. hippocampal volumes, atrophy of the MB and parahippocampal white matter, were strongly associated with especially cognitive and memory problems. Furthermore, hippocampal volumes were significantly smaller in children with MB atrophy compared to normal MB. Even after correction for grey matter, white matter, cerebellar and lateral ventricle volumes, hippocampal volume and MB atrophy remained significantly associated with IQ measures, verbal long-term memory and visual-spatial long-term memory. Finally, larger hippocampal volumes were associated with higher FA values throughout the white matter, also normal MB were associated with higher FA values compared to children with MB atrophy.

Hippocampal volume and MB atrophy were strongly associated with neurocognitive outcome and episodic memory at 10 years of age. In non-cooled infants, the association between of hippocampal volumes and episodic memory functioning has been described previously^8,9^. The present study confirmed those findings in cooled infants. The MB are also known to be important for episodic memory^26^. Recently, Molavi et al*.* described that 13.2% of the infants with HIE had an abnormal signal intensity on their T2-weighted MRI^10^. The strong association between subsequent atrophy of the MB and long-term problems has not been described previously in children with HIE. The association between smaller MB volumes and cognitive and memory impairments has recently been shown in adolescents with a Fontan procedure for a single ventricular heart disease^27^. The MB are easy to score without additional software or post-processing and atrophy is significantly associated with neurodevelopmental outcome, hippocampal volumes and FA values, making it an easy indicator for neurodevelopmental outcome. Atrophy of the MB is already visible at three months after birth^10^. So, the MB are important to routinely assess in infants with HIE and should be added to the available scoring systems.

Besides the hippocampus and MB, the anterior thalamus and fornix are also part of the Papez circuit^11^. The thalamus was not associated with outcome in this study, but we were not able to segment the anterior thalamus separately. Also, the fornix could not be reliably segmented with Freesurfer. However, we did find a significant association between normal MB and higher FA values in the fornix and between larger hippocampal volumes and higher FA values in the fornix.

Other brain structures should also be taken into account when assessing brain injury in children with HIE during follow-up, since perinatal asphyxia leads to global hypoxic-ischemia of the brain^28^. For example, in the TBSS analyses FA values in the anterior corpus callosum were associated with PIQ and visual-spatial long-term memory, and the volume of the corpus callosum was also associated with processing speed and verbal long-term memory (although not significant after correction for multiple comparisons). Furthermore, the amygdala and parahippocampal white matter were associated with hippocampal volumes and might be related to neurodevelopmental outcome in a larger cohort.

Interestingly, FA values conducted with TBSS were only positively associated with PIQ and visual-spatial long-term memory. In the neonatal period, lower FA values in e.g. the posterior limb or the internal capsule and corpus callosum are known to be predictive for long-term outcome^29^. Literature about FA values at school-age in children with a history of HIE following perinatal asphyxia is scarce. Laporta-Hoyos et al*.* found that FA values were related to IQ and executive functioning in adults with dyskinetic CP, but not in the controls^30^. Attention and information processing were not associated with FA values in both groups^30^. The authors concluded that FA values are only associated with cognition in the presence of severe brain injury^30^. This might explain the absence of an association between FA and IQ and memory in our population, since brain injury at neonatal age and at 10 years of age was only mild to moderate.

Our study also showed that, although therapeutic hypothermia has proven to be neuroprotective, neurodevelopmental problems at school-age are still prevalent. In the short-term, therapeutic hypothermia reduces death, epilepsy and CP in infants with HIE^6,31^. In our cohort, CP was also less often diagnosed in the screened population that was cooled compared to the non-cooled cohort. However, the actual incidence of CP and death could not be compared due to the study design. The comparisons between the HT and non-HT group should therefore be interpreted with caution, because this is not a randomized controlled trial. We noted that the lactate levels were higher and Apgar scores were lower in the HT group compared to the non-HT group which suggests that the HT group suffered more severe asphyxia. MB atrophy was also more frequent at the age of 10 years in the HT group. This might explain the finding that therapeutic hypothermia was associated with a lower verbal long-term memory (immediate recall).

Nevertheless, we were able to show that memory and cognitive problems can develop at school-age even in children who were treated with therapeutic hypothermia. Shankaran et al*.* described that the incidence of death and CP were lower in 6- to 7-year-old children with HIE that were cooled compared to controls, but cognitive, attention and visuospatial function did not significantly improve^32^. The TOBY trial also revealed a better survival with an IQ above 85, but similar intelligence, memory, learning, sensorimotor and visuospatial processing between cooled and non-cooled children at 6–7 years of age^5^. Furthermore, quality of life at 6–7 years of age was comparable in the cooled and non-cooled group^33^.

Memory and behavioural problems are often not recognized until school-age, emphasizing the need for long-term follow-up. Hayes et al*.* confirmed that children with HIE show more problems at the age of 42 months and above in domains of attention, memory and behaviour than was expected based on the 2 year assessment with the Bayley-III^34^. Furthermore, it has been reported that one third of the cooled infants without CP still experienced motor problems at the age of 6 to 8 years that were not recognized during the regular 18-month examination^35^. Clinicians should realize, that even with a normal 18–24 month follow-up, deficits can still become apparent at school-age.

This study has several limitations. First of all, as mentioned before, the study design is not randomized and does not allow us to draw conclusions about the effect of therapeutic hypothermia at school-age. Also, no data of healthy controls were available for comparison. Secondly, a bias in screening and inclusion criteria might have resulted in slightly different groups. Lactate and Apgar scores suggest that the HT group was more severely affected than the non-HT group. Thompson scores were not conducted in the non-HT group, but the patient files were studied to ascertain that infants would have fulfilled the hypothermia criteria. Furthermore, numbers about neonatal death are difficult to compare, since in the hypothermia era all infants born in level II hospitals that fulfilled hypothermia criteria were transferred to the level III hospitals and before 2008 one cannot exclude that infants who were in a very poor neurological condition were not transferred to the level II hospital and may have died in the level II hospitals. Lastly, the brain volumes were highly correlated. This made it impossible to include different brain volumes in a model and determine which brain volume is most associated with different outcome measures. This may be solved by principal component analysis in future larger studies.

This study has multiple implications for clinical care and follow-up of infants with HIE. First of all, therapeutic hypothermia does decrease neonatal death, CP, and epilepsy, but might not sufficiently protect the brain to prevent cognitive and memory problems at school-age. Different add-on therapies are currently being investigated in large trials^36^, which hopefully help to further improve long-term neurodevelopmental outcome. Furthermore, early atrophy of the MB is strongly predictive for long-term outcome and easy to assess. This might be an early indicator for long-term cognitive outcome, also in infants who are treated with therapeutic hypothermia. Finally, this study underlines the importance of long-term follow-up into childhood, and maybe even adulthood, to assess definitive outcomes of infants with HIE.

In summary, children with HIE who were cooled still suffer from neurocognitive and memory problems at 10 years of age. Atrophy of the MB, hippocampus and parahippocampal white matter was strongly associated with neurodevelopmental problems. Neonatal abnormalities of the MB in the acute phase of HIE can be a biomarker to predict atrophy of the MB, which appears to be associated with neurodevelopmental difficulties at school-age.

## Supplementary Information

Supplementary Information

## Data Availability

The datasets generated and/or analysed during the current study are available from the corresponding author on reasonable request.

## Abbreviations

BOT-2: Bruininks–Oseretsky test of motor proficiency second edition
BRIEF: Behaviour rating inventory of executive function
CBCL: Children’s behaviour checklist
CP: Cerebral palsy
DWI: Diffusion weighted imaging
DTI-TK: DTI-ToolKit
FA: Fractional anisotropy
FLAIR: Fluid attenuated inversion recovery
HIE: Hypoxic-ischemic encephalopathy
HT: Therapeutic hypothermia
IQ: Intelligence quotient
MB: Mammillary bodies
PIQ: Performance intelligence quotient
TBSS: Tract-based spatial statistics
TBV: Total brain volume
TIQ: Total intelligence quotient
VIQ: Verbal intelligence quotient
WISC: Wechsler intelligence scale for children
