# Supplementary material for: Mammillary body atrophy and other MRI correlates of school-age outcome following neonatal hypoxic-ischemic encephalopathy
Source: Sci Rep. 2021 Mar 3;11:5017. doi: 10.1038/s41598-021-83982-8 (PMC7930036; doi:10.1038/s41598-021-83982-8)
Supplement: Supplementary file 1 — Supplementary Information [file 41598_2021_83982_MOESM1_ESM.docx]

**Supplementary material**

**Mammillary body atrophy and other MRI correlates of school-age outcome following neonatal hypoxic-ischemic encephalopathy**

**Authors:**

Kim V. Annink, MD ^1^,Linda S. de Vries, MD, PhD ^1^, Floris Groenendaal, MD, PhD ^1^, Rian M.J.C. Eijsermans, MD ^2^, Manouk Mocking, MD ^3^, Monique M.J. van Schooneveld, MD, PhD ^3^, Jeroen Dudink, MD, PhD ^1^, Henrica L.M. van Straaten, MD, PhD ^4^, Manon J.N.L. Benders, MD, PhD ^1^, Maarten Lequin, MD, PhD ^5#^, Niek E. van der Aa, MD, PhD ^1#^*

**Supplemental table 1:** mammillary bodies on the neonatal MRI compared to the MRI at 10 years of age

| Neonatal MB score | Normal MB at 10y | Atrophy MB at 10y |
| --- | --- | --- |
| Normal, n (%) | 10 (91) | 1 (9) |
| Equivocal, n (%) | 9 (90) | 1 (10) |
| Abnormal, n (%) | 4 (24) | 13 (76) |

**Supplementary table 2:** univariate linear associations between MB atrophy and neurodevelopmental outcome with a p-value < 0.05.

| Outcome measure | MB atrophy | B-value | R^2^ value | p-value |
| --- | --- | --- | --- | --- |
| TIQ | MB atrophy | -9.6 | 0.337 | <0.001** |
| VIQ | MB atrophy | -7.1 | 0.211 | 0.002** |
| PIQ | MB atrophy | -10.7 | 0.356 | <0.001** |
| Processing speed | MB atrophy | -7.1 | 0.162 | 0.007 |
| Verbal long-term memory, immediate recall | MB atrophy | -1.7 | 0.298 | <0.001** |
| Verbal long-term memory, delayed recall | MB atrophy | -1.3 | 0.225 | 0.001** |
| Visual-spatial long-term memory, direct recall | MB atrophy | -9.1 | 0.315 | <0.001** |
| Visual-spatial long-term, delayed recall | MB atrophy | -9.7 | 0.446 | <0.001** |

**Supplementary table 3:** univariate linear associations of the segmented brain volumes and outcome measures. Of the 80 segmented volumes, only volumes an association with p-value <0.05 are shown in this table.

| Outcome measure | Relative volume (%) | B-value | R^2^ value | p-value |
| --- | --- | --- | --- | --- |
| TIQ | Hippocampus | 65.8 | 0.112 | 0.032 |
|  | Putamen | -85.7 | 0.161 | 0.009 |
|  | Caudal anterior cingulate white matter | -122.8 | 0.147 | 0.015 |
|  | Rostal anterior cingulate white matter | -188.3 | 0.191 | 0.005 |
|  | Superior frontal white matter | 29.8 | 0.129 | 0.023 |
|  | Insula white matter | -48.6 | 0.146 | 0.015 |
| VIQ | Putamen | -60.5 | 0.106 | 0.046 |
|  | Superior frontal white matter | 26.0 | 0.133 | 0.026 |
| PIQ | Hippocampus | 88.8 | 0.190 | 0.006 |
|  | Putamen | -76.7 | 0.119 | 0.031 |
|  | Caudal anterior cingulate white matter | -116.1 | 0.124 | 0.030 |
|  | Insula white matter | -49.2 | 0.136 | 0.023 |
|  | Rostal anterior cingulate cortex | -100.6 | 0.115 | 0.040 |
|  | Frontal cortex | -124.3 | 0.109 | 0.046 |
| Processing speed | Hippocampus | 97.2 | 0.234 | 0.002** |
|  | Amygdala | 221.1 | 0.136 | 0.019 |
|  | Thalamus | 54.8 | 0.100 | 0.046 |
|  | Corpus callosum | 160.6 | 0.108 | 0.038 |
|  | Caudal anterior cingulate white matter | -115.5 | 0.114 | 0.036 |
|  | Parahippocampal white matter | 261.3 | 0.292 | <0.001** |
|  | Caudal anterior cingulate cortex | -153.8 | 0.279 | 0.001** |
|  | Parahippocampal cortex | 157.2 | 0.160 | 0.013 |
|  | Pericalcarina cortex | 67.0 | 0.113 | 0.039 |
|  | Rostal anterior cingulate cortex | -96.7 | 0.136 | 0.023 |
| Verbal long-term memory, immediate recall | Hippocampus | 19.0 | 0.284 | <0.001** |
|  | Corpus callosum | 35.7 | 0.169 | 0.008 |
|  | Isthmus cingulate white matter | 22.3 | 0.141 | 0.018 |
|  | Parahippocampal white matter | 41.5 | 0.226 | 0.002 |
|  | Superior temporal white matter | 12.9 | 0.158 | 0.012 |
|  | Supra marginal white matter | 8.9 | 0.143 | 0.018 |
|  | Caudal anterior cingulate cortex | -22.1 | 0.176 | 0.009 |
|  | Lateral occipital cortex | -5.9 | 0.185 | 0.007 |
|  | Posterior cingulate cortex | -14.5 | 0.128 | 0.028 |
| Verbal long-term memory, delayed recall | Hippocampus | 11.8 | 0.135 | 0.020 |
|  | Corpus callosum | 27.0 | 0.120 | 0.029 |
|  | Brain stem | 6.8 | 0.125 | 0.025 |
|  | Isthmus cingulate white matter | 22.5 | 0.178 | 0.008 |
|  | Parahippocampal white matter | 38.0 | 0.234 | 0.002 |
|  | Lateral occipital cortex | -4.6 | 0.143 | 0.019 |
|  | Medial orbitofrontal cortex | -9.8 | 0.109 | 0.043 |
|  | Frontal cortex | -24.1 | 0.149 | 0.017 |
| Visual-spatial working memory | Precuneus cortex | 18.6 | 0.111 | 0.047 |
| Verbal working memory | Precuneus white matter | 7.9 | 0.118 | 0.033 |
|  | Superior parietal white matter | 5.6 | 0.150 | 0.015 |
|  | Supra marginal white matter | 7.6 | 0.101 | 0.048 |
|  | Frontal white matter | -104.2 | 0.109 | 0.040 |
| Visual-spatial long-term memory, direct recall | Hippocampus | 67.1 | 0.133 | 0.022 |
|  | Inferior temporal white matter | 56.1 | 0.127 | 0.028 |
|  | Middle temporal white matter | 57.0 | 0.128 | 0.027 |
|  | Superior frontal white matter | 26.4 | 0.126 | 0.029 |
|  | Lateral orbitofrontal cortex | -42.3 | 0.161 | 0.014 |
|  | Pars orbitalis cortex | -90.8 | 0.132 | 0.027 |
|  | Precentral cortex | -24.6 | 0.110 | 0.045 |
|  | Frontal cortex | -159.7 | 0.200 | 0.006 |
| Visual-spatial long-term, delayed recall | Hippocampus | 74.4 | 0.219 | 0.003 |
|  | Entorhinal white matter | 193.3 | 0.119 | 0.036 |
|  | Parahippocampal white matter | 136.0 | 0.124 | 0.033 |
|  | Precentral white matter | -28.0 | 0.112 | 0.042 |
|  | Superior frontal white matter | 25.9 | 0.129 | 0.029 |
|  | Isthmus cingulate cortex | 82.7 | 0.110 | 0.048 |
|  | Lateral orbitofrontal cortex | -32.8 | 0.126 | 0.034 |
|  | Pars orbitalis cortex | -72.5 | 0.109 | 0.049 |
|  | Precentral cortex | -30.3 | 0.215 | 0.004 |
|  | Frontal cortex | -114.5 | 0.133 | 0.029 |

* After correction for multiple comparison, p-values < 0.0025 are statistically significant for the segmentations and p-values of <0.00125 for the parcellations of cortex and white matter.
